# Fallow Land Enhances Carbon Sequestration in Glomalin and Soil Aggregates Through Regulating Diversity and Network Complexity of Arbuscular Mycorrhizal Fungi Under Climate Change in Relatively High-Latitude Regions

**DOI:** 10.3389/fmicb.2022.930622

**Published:** 2022-07-04

**Authors:** Yurong Yang, Wenbo Luo, Jiazheng Xu, Pingting Guan, Liang Chang, Xuefeng Wu, Donghui Wu

**Affiliations:** ^1^Key Laboratory of Vegetation Ecology, Ministry of Education, School of Life Sciences, Northeast Normal University, Changchun, China; ^2^State Environmental Protection Key Laboratory of Wetland Ecology and Vegetation Restoration, School of Environment, Northeast Normal University, Changchun, China; ^3^Key Laboratory of Wetland Ecology and Environment, Northeast Institute of Geography and Agroecology, Chinese Academy of Sciences, Changchun, China; ^4^School of Tourism and Service Management, Chongqing University of Education, Chongqing, China

**Keywords:** climate warming, land-use conversion, soil structure, symbiotic fungi, glomalin, carbon sequestration

## Abstract

Soil aggregation and aggregate-associated carbon (C) play an essential function in soil health and C sequestration. Arbuscular mycorrhizal fungi (AMF) are considered to be primary soil aggregators due to the combined effect of extraradical hyphae and glomalin-related soil proteins (GRSPs). However, the effects of diversity and network complexity of AMF community on stability of soil aggregates and their associated C under long-term climate change (CC) and land-use conversion (LUC) in relatively high-latitude regions are largely unexplored. Therefore, an 8-year soil plot (with a 30-year cropping history) transplantation experiment was conducted to simulate CC and LUC from cropland to fallow land. The results showed that *Glomus*, *Paraglomus*, and *Archaeospora* were the most abundant genera. The diversity of AMF community in fallow land was higher than cropland and increased with increasing of mean annual temperature (MAT) and mean annual precipitation (MAP). Fallow land enhanced the network complexity of AMF community. The abundance families (Glomeraceae and Paraglomeraceae) exhibited higher values of topological features and were more often located in central ecological positions. Long-term fallow land had a significantly higher hyphal length density, GRSP, mean weight diameter (MWD), geometric mean diameter (GMD), and C concentration of GRSP (C-GRSP) than the cropland. The soil aggregate associated soil organic carbon (SOC) was 16.8, 18.6, and 13.8% higher under fallow land compared to that under cropland at HLJ, JL, and LN study sites, respectively. The structural equation model and random forest regression revealed that AMF diversity, network complexity, and their secreted GRSP mediate the effects of CC and LUC on C-GRSP and aggregate-associated SOC. This study elucidates the climate sensitivity of C within GRSP and soil aggregates which response symmetry to LUC and highlights the potential importance of AMF in C sequestration and climate change mitigation.

## Introduction

Human activities are majorly responsible for an increase in worldwide average temperatures, and it is expected that the mean surface temperature across the globe will be increased by 3 and 4°C at the end of the 21st century relative to the reference period of 1986–2005 (IPCC, 2018). Climate change (CC) can cause soil surface aridity at global scale due to reduced precipitation in the subtropics and increased evaporative demand associated with higher vapor pressure deficit (Dai et al., 2018). Land-use change reflected in land-use conversion (LUC) and land-cover change is another main component of global environmental change. The increased frequency of drought, temperature fluctuations, and habitat loss by CC and LUC exhibited adverse effect on soil microbial activities, nutrient processing, and crop productivity (Raza et al., 2019; Van Sundert et al., 2021). However, CC and LUC are expected to have different effects in various geographic areas. Previous studies indicated that CC exerted beneficial effects on biodiversity at high latitudes while simultaneously decreased biodiversity at low latitudes (Dyderski et al., 2018; McElwee, 2021). Yet, it is still unclear the interactive effects of CC and LUC on the function and services of soil ecosystem in relatively high-latitude areas.

Soil aggregates are the basic components of soil structure, and the stability of soil aggregates has made great contribution to the function and services of soil ecosystem such as plant production, water-holding capacity, nutrient cycling, and carbon (C) sequestration (Wilson et al., 2009; Gupta and Germida, 2015). A stable soil aggregate can reduce C loss and decrease the rate of microbial decomposition through coating and isolation effects (Sekaran et al., 2021). For example, large macro-aggregates can bind much higher soil organic carbon (SOC), but was more sensitive to LUC compared with micro-aggregates (Sekaran et al., 2021). Previous studies indicated higher aggregate stability and C concentration of stable macro-aggregates in natural ecosystems compared to cultivated ecosystems (Pang et al., 2020; Sekaran et al., 2021). Herein, soil aggregation has been widely considered as an important process of C cycling, and it is highly sensitive to environmental changes (Tisdall and Oades, 1982; Wang et al., 2019; Du et al., 2022).

The process of soil aggregation is regulated by a range of abiotic and biotic factors as well as their interactions. As one of the most important biotic factors, arbuscular mycorrhizal fungi (AMF) play a crucial role in soil aggregate formation and stabilization (Rillig et al., 2002). Glomalin, measured as glomalin-related soil protein (GRSP), is a hyphal glycoprotein substance that can be released by AMF into the soil during hyphal turnover and after the death of the fungi (Rillig, 2004). According to the soil aggregation hierarchical theory proposed by Tisdall and Oades (1982), the primary soil particles are initially bonded by GRSP and then held together by AMF hyphae so as to form macro-aggregates to promote the stability of soil aggregates (Steinberg and Rillig, 2003). Although AMF have a crucial role in multiple ecosystem functions and soil processes *via* several pathways, the effects of AMF species diversity on soil aggregate stability are largely unexplored (Wilson et al., 2009). In addition, the interactions among AMF species can regulate the structure and function of AMF communities through influencing their resource competition (Ma et al., 2020). Co-occurrence network analysis is an important method for uncovering the structure of microbial communities that provides new insights into potential interactions among microbial species (Barberán et al., 2012). The results from long-term field experiments showed that both soil aggregation and C sequestration were highly correlated with AMF abundance (Wilson et al., 2009). However, the contributions of AMF diversity, community composition, and network complexity to soil aggregation and C sequestration under CC and LUC remain largely unknown.

The influence of CC and LUC on soil aggregation is a quite complex process. The most important direct effects are the aggregate destruction role of raindrops and mechanical force, whereas the indirect impacts are induced by the changes in the soil properties and GRSP secreted by AMF. LUC, such as land conversion of cropland to grasslands or forage land, exerts direct effects on aggregate-forming processes in which macro-aggregates are sensitive to these short-term disturbances (Qian et al., 2018). Meanwhile, the growth parameters, diversity, community composition, and co-occurrence topological features of AMF were also impacted by CC and LUC. Temperature manipulation studies have indicated that the richness of the AMF community responds to warming in various ways, with neutral (Heinemeyer et al., 2004), positive (Yang et al., 2013), and negative effects (Che et al., 2019) detected. GRSP was sensitive to LUC, and the higher level of GRSP was observed in pasture and agroforestry systems compared with the forest (Matos et al., 2022). However, understanding of the relationships among AMF diversity, network complexity, GRSP, and soil aggregate stability and their response to CC and LUC in relatively high-latitude regions are limited.

Moreover, the C concentration of GRSP (C-GRSP) was found to be 2–24 times larger than that of soil humus, accounting for 30–40% of SOC, indicating a direct effects of GRSP on C storage in soils (Hossain, 2021). Aggregate size is also a crucial variable influencing C sequestration and decomposition. Generally, macro-aggregates contain higher concentrations of C than micro-aggregates. However, macro-aggregates are more sensitive to LUC and have a significantly faster turnover rate compared with micro-aggregates. Thus, it is difficult to predict exactly how CC and LUC affect the C-GRSP and aggregate-associated SOC, which is important for understanding the C cycling in soils, especially in relatively high-latitude areas. The objective of this study was to (1) assess the response of AMF diversity, network complexity, growth parameters, and their secreted GRSP to CC and LUC, (2) determine the effects of CC and LUC on C-GRSP and soil aggregate-associated SOC, and (3) reveal the mechanisms of CC and LUC on C-GRSP and soil aggregate-associated SOC in agricultural ecosystem in relatively high-latitude regions.

## Materials and Methods

### Study Sites

An 8-year soil plot transplantation experiment was conducted at three experimental stations of the Chinese Academy of Sciences across Heilongjiang, Jiling, and Liaoning provinces in northeast China (Supplementary Figure 1): the Sanjiang Field Experimental Station is located in Heilongjiang Province (HLJ); the Changchun Agricultural Experimental Station is located in Jilin Province (JL); and the Liaozhong Agricultural Experimental Station is located in Liaoning Province (LN). This region was approximately 2,100 km long with a temperate continental monsoon climate. Along this region, the mean annual temperature (MAT) ranged from 3.3–8.5°C, and the mean annual precipitation (MAP) ranged from 579.9 to 651.2 mm. The detailed information about the geographical location and the basic climate parameters of these three stations is shown in Supplementary Table 1.

In April 2011, 30 original soil plots (1 m in length, 1 m in width, and 1 m in depth) were collected from 30-year-old soybean fields at HLJ site. The soil plots were excavated in five layers, and each layer (20 cm) was thoroughly mixed and restacked to simulate the original soil stratification in the PVC boxes (1 m in length, 1 m in width, and 1 m in depth) (Supplementary Figure 2). Finally, 10 PVC boxes were kept *in situ* (HLJ) as a control, and the other 20 PVC boxes were transported to the JL and LN sites separately (10 PVC boxes at each site) to simulate CC gradient (Supplementary Figure 1). At each study site (10 PVC boxes), there were five PVC boxes for cropland treatment and the other five PVC boxes for fallow-land treatment.

### Experimental Design and Sampling

The experiment was a 3 × 2 factorial scheme (three climate conditions and two land-use types) that was established in a randomized complete block design (RCBD) with five replicates. The CC had three levels (HLJ site with a MAT of 3.3°C, JL site with a MAT of 6.4°C, and LN site with a MAT of 8.5°C), whereas the LUC had two levels (cropland and fallow land). Thus, each study site contains cropland treatment and fallow-land treatment. In cropland, the seeds of soybean [*Glycine max* (L.) Merr.] were sown in three ridges (15 plants per ridge) per plot on the same day every year in spring (May 20). Soybean seedlings were harvested and removed at all sites on the same day each year in autumn (August 10). In contrast, plants grew freely, and the soil was undisturbed throughout the fallow period in fallow land. The plant diversity in fallow land was significantly higher than that in cropland, and the most dominant plants were *Sonchus arvensis* L., *Chenopodium album* L., *Commelina communis* L., and *Polygonum lapathifolium* L.

In July 2018, we used handheld coring devices to collect soil samples (0–20 cm) (10 soil samples with five replicates per study site). Soil samples were passed through a sieve (10 mesh) to remove large stones and plant debris. Thereafter, each sample was divided into three subsamples. Approximately 10 g soil samples were transferred to 10-ml tubes, immediately frozen in a tank with liquid nitrogen, and stored at –80°C prior until soil microbial DNA extraction. Approximately 40 g soil samples were stored in Ziploc bags and kept at 4°C in cool boxes during transport to the laboratory and then stored in a refrigerator at –20°C before the determination of ammonium (NH_4_^+^-N) and nitrate (NO_3_^–^-N) concentrations. Approximately 100 g soil samples were air-dried at room temperature prior to the measurement of soil pH, organic carbon (SOC), dissolved organic carbon (DOC), total nitrogen (TN), available nitrogen (AN), total phosphorus (TP), and available phosphorus (AP). Furthermore, undisturbed soil samples were collected using a 6 cm × 5 cm (diameter × height) metal ring and stored in plastic boxes to evaluate soil aggregate stability.

### Soil Properties

Soil pH was measured in suspension of 1:2.5 (m/v) soil/water ratio using a pH meter (FE28, Mettler Toledo, Zurich, Switzerland). SOC was assessed using a TOC analyzer (Vario MICRO cube, Elementar Ltd., Germany). Soil TN was qualified according to the micro-Kjeldahl procedure (Jackson, 1956). Soil DOC was determined by high-temperature catalytic oxidation with a TOC analyzer (TOC-VCPH, Shimadzu Co., Ltd., Kyoto, Japan) (Wu et al., 2015). Soil NH_4_^+^-N and NO_3_^–^-N were determined with a continuous-flow analyzer (Alliance Instruments, Frepillon, France). Soil AN was qualified according to the alkaline hydrolysis diffusion method (Khan et al., 1997). Soil TP was measured by the molybdenum blue colorimetric method (Sommers and Nelson, 1972). Soil AP was extracted with 0.5 M NaHCO_3_ and then assessed by a continuous-flow analyzer (SAN^++^; Skalar, Breda, Holland). The distribution and stability of water-stable aggregation (WSA) were assessed based on a wet-sieving apparatus (Karlen et al., 2013) and also summarized in the Supporting Information (Method 1). After the removal of any coarse organic particles, the dried aggregate fractions were ground in a mortar and pestle. The aggregate-associated SOC was determined based on the Walkley–Black chromic acid wet oxidation method (Walkley and Black, 1934). Total GRSP (T-GRSP) and easily-extractable GRSP (EE-GRSP) were extracted from soil samples using a method described by Wright and Upadhyaya (1996), and the concentrations of T-GRSP and EE-GRSP were measured with a spectrophotometer (Bradford, 1976).

### AMF Growth Parameters

Plant roots were washed by tap water, cleared with 10% KOH, and stained with 0.05% trypan blue (Phillips and Hayman, 1970). The mycorrhizal colonization, spore density, and hyphal length density were qualified according to the methods described by Biermann and Linderman (1981); Gerdemann and Nicolson (1963), and Jakobsen et al. (1992), respectively. The detailed information is shown in the Supporting Information (Method 2).

### DNA Extraction, Pyrosequencing, and Data Processing

Detailed description of DNA extraction, 16S rRNA gene amplification primers (Supplementary Table 2), and sequence processing were reported previously (Wang et al., 2021) and also summarized in the Supporting Information (Method 3). The Illumina sequencing raw read data deposited in the Sequence Read Archive (SRA) are available in the NCBI SRA portal with PRJNA797899, BioProject ID.

### Co-occurrence Network Analysis

Co-occurrence network analysis has been increasingly performed in microbial ecology in the recent years to better understand microbial community structure and to characterize potential intracommunity interactions among species. A total of eight co-occurrence networks of the soil AMF communities from cropland and fallow land at three study sites were constructed based on OTU abundance. We calculated the Spearman’s correlation coefficient in the soil AMF community in R with the package Hmisc (Harrell and Frank, 2008). Only the robust correlations with |*r*| ≥ 0.6 and *p* < 0.01 were kept to construct networks. Thereafter, multiple *p*-values were adjusted according to the false discovery rate (FDR) with the Benjamini controlling procedure (Benjamini et al., 2006). The co-occurrence networks were visualized, and the topological features (i.e., numbers of nodes and edges, modularity, network diameter, clustering coefficient, graph density, and average path length) were estimated by the igraph R package (Csárdi and Nepusz, 2006). The modular structure of the microbial community was evaluated *via* the modularity index (Lambiotte et al., 2015). The nodes with a high degree (top 1% of interactions) were regarded as possible keystone species in the networks (Banerjee et al., 2018). Moreover, 1,000 Erdös-Rényi random networks with the same number of nodes and edges as the real networks were generated for comparison with the real networks by the igraph R package (Erdös and Rényi, 1960).

### Statistical Analysis

The richness (Sobs, Chao, and ACE indices) and diversity (Shannon index) of AMF were determined using Mothur (Schloss et al., 2011). One-way analysis of variance (ANOVA) was performed to analyze the effects of CC and LUC on AMF community α-diversity (Sobs, Ace, Chao, and Shannon indices), AMF growth index (MC, SPD, and HLD), GRSP (T-GRSP and EE-GRSP), and soil aggregate stability (MWD and GMD). Significant differences among treatments were evaluated using Tukey’s HSD test at *p* < 0.05. Principal coordinate analysis (PCoA) with the Bray–Curtis distance was performed to determine changes in AMF communities. Non-parametric MANOVA (Bray–Curtis matrix, 999 permutations) using the adonis function of R was conducted to assess the significant differences in AMF community composition among different treatments. A *post hoc* pairwise adonis test was then performed using the “pairwise.adonis” function implemented in the R package pairwiseAdonis (Martinez Arbizu, 2017). The correlations between AMF community composition and environmental variables were estimated by distance-based redundancy analysis (db-RDA) in the R package VEGAN (Oksanen et al., 2007). Variation partitioning analyses (VPAs) were further employed to quantify the proportional contributions of CC, LUC, and soil properties to AMF community structure. A structural equation model (SEM) was performed to evaluate both the direct effects and indirect effects of CC, LUC, soil properties, AMF diversity, AMF growth index, and GRSP on the stability of soil aggregates. Before the SEM procedure, we separately reduced the number of variables for CC, LUC, soil properties, AMF diversity, AMF growth index, GRSP, and stability of soil aggregates through principal component analysis (PCA) (Veen et al., 2010). For each group with multiple variables, the first principal component (PC1) explained 66.19–99.59% of the total variance that would be applied in the subsequent SEM analysis (Supplementary Table 3). SEM analysis was performed using Amos 21.0 software (Amos Development Corporation, Chicago, IL, United States) based on the results of the correlation analysis. The fitness of the model was determined by the chi-square test, the root mean-square residual (RMR), goodness-of-fit index (GFI), comparative fit index (CFI), and relative fit index (RFI) (Liu et al., 2017). The PCAs were conducted using the vegan package in R software. The random forest model was selected to identify the most important environmental variables that affect the stability of soil aggregates using the randomForest package (Liaw and Wiener, 2002). All data analyses and visualization, except SEM, were performed in R 3.4.1 (R Core Team, 2017).

## Results

### Overall Pyrosequencing Information and α-Diversity of AMF

**FIGURE 1:**
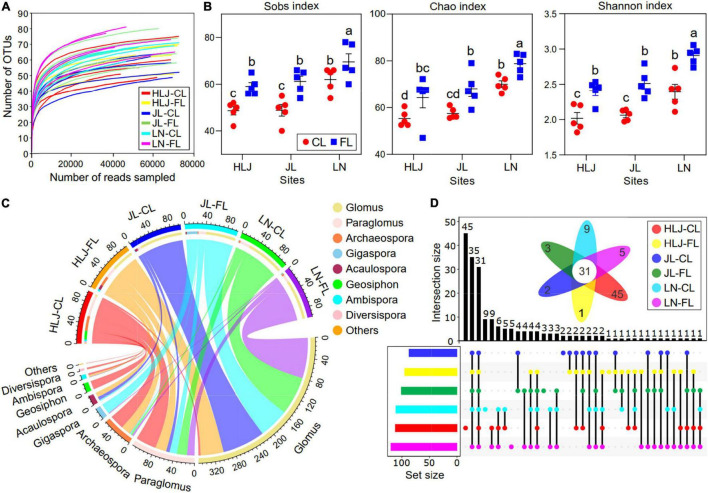
Composition and diversity of the AMF community under CC and LUC. **(A)** Rarefaction curves of the observed OTU number of AMF; **(B)** box plots representing α-diversity indices at the OTU level, including Sobs, Chao, and Shannon indices under different treatments; **(C)** circos diagram depicting the top AMF composition at the genus level with treatment; and **(D)** upset plots showing the relationships of AMF genus in different treatments. The colored bars on the left panel indicate the number of genera that are uniquely shared in each sample. The numbers above the bars show the number of common genera among the groups of samples marked below the bars. A Venn diagram of shared and unique genera is shown. CL, cropland; FL, fallow land.

A total of 2,850,427 sequences (ranging from 41,612–74,080 reads per sample) with a minimal length 236 bp were detected from all samples. After rarefying, 199 AMF OTUs were detected, which could be assigned to 9 genera and 48 species. All rarefaction curves to be saturated with increased sequencing amounts (Figure 1A) and the coverage exceeded 99%, which indicated that the vast majority of AMF had been sequenced in all samples. α-diversity in AMF communities was calculated by three methods (Figure 1B). The α-diversity of AMF communities was significantly influenced by CC and LUC, but not by their interaction (Figure 1B and Supplementary Table 4). Specifically, the Sobs, Chao, and Shannon indices were significantly higher in fallow land compared to cropland, and they increased with increase in mean annual temperature (MAT, *p* < 0.001) and mean annual precipitation (MAP, *p* < 0.001). *Glomus* was the most abundant genus (58.50% of the total sequence) followed by *Paraglomus* (18.88%) and *Archaeospora* (9.60%) (Figure 1C). The dominant genera varied considerably among the different soil samples. *Glomus* was more enriched at JL and LN site, whereas *Paraglomus* and *Archaeospora* were more enriched at HLJ site (Figure 1C). The largest number of unique OTUs was obtained from cropland at HLJ site with 45 OTUs (Figure 1D). In descending order, the unique OTUs were 9, 5, 3, 2, and 1 OTUs in cropland at LN site, fallow land at LN site, fallow land at JL site, cropland at JL site, and fallow land at HLJ site, respectively.

The core AMF communities were defined as the shared taxa of all soil samples as displayed by flower diagrams. A total of thirty-one core OTUs were identified in all soil samples (Figure 1D); these core OTUs accounted for 15.58% of the relative abundance of the AMF community.

### AMF Community Structure and Driving Factors

**FIGURE 2:**
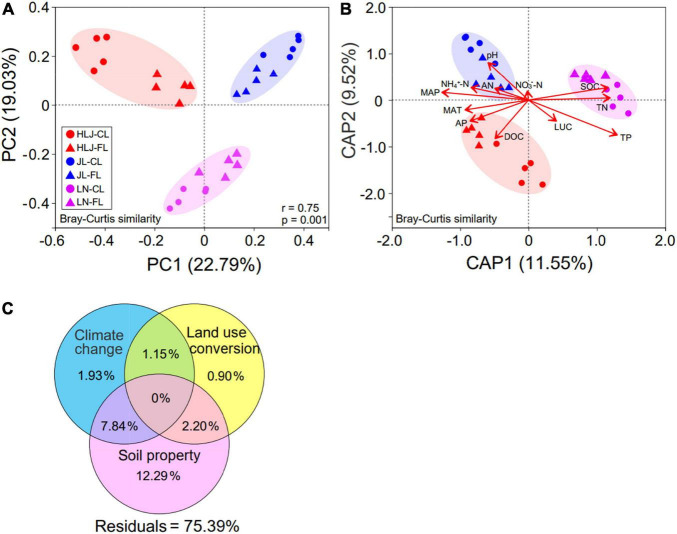
AMF community structure and influencing factors. **(A)** Principal coordinate analysis (PCoA) of AMF community structure (Bray–Curtis dissimilarity) under climate change and land-use conversion. The ellipses represent standard deviations; **(B)** distance-based redundancy analysis (db-RDA) of AMF community structure (Bray–Curtis dissimilarity) in response to CC, LUC and soil properties. Variables and environmental vector correlation plots show the directions and strengths of the relationships between two environmental factors. Axis legends represent the percentage of variation explained by the axis; and **(C)** variation partitioning analysis (VPA) of the effects of climatic climate, LUC, and soil properties on AMF community structure. CL, cropland; FL, fallow land; MAT, mean annual temperature; MAP, mean annual precipitation; DOC, dissolved organic carbon; SOC, soil organic carbon; AN, available nitrogen; AP, available phosphorus; TP, total phosphorus; TN, total nitrogen. NO_3_^–^-N, nitrate nitrogen; NH_4_^+^-N, ammonium nitrogen.

The PCoA plot (Figure 2A) and permutational multivariate analysis of variance (PERMANOVA) suggested that CC (*F* = 11.76, *R*^2^ = 0.386, *p* = 0.001), LUC (*F* = 5.79, *R*^2^ = 0.095, and *p* = 0.001), and their interaction (*F* = 11.76, *R*^2^ = 0.386, and *p* = 0.001) significantly impacted the AMF community composition (Supplementary Table 5). CC showed a greater impact on AMF communities than LUC based on the associated *F* values.

A canonical analysis of principal coordinates (CAP) was performed to identify soil properties and climatic variables (Supplementary Table 5) that explained variations in the AMF community structure. Among all environmental attributes, TP (*R*^2^ = 0.545, *p* = 0.001) and MAP (*R*^2^ = 0.417, *p* = 0.002) appeared to be the most important environmental factors in driving AMF community composition (Figure 2B and Supplementary Table 5). Furthermore, the AMF community was significantly correlated with SOC, DOC, AP, TN, pH, and MAT, but was not correlated with LUC, AN, NO_3_^–^-N, or NH_4_^+^-N (Figure 2B and Supplementary Table 5). To quantify the contribution of soil properties, CC, and LUC to AMF community composition, we performed a variation partitioning analysis. A total of 24.61% of the variation was explained by these three environmental variables (Figure 2C). Soil properties and CC independently explained 12.29 and 1.93%, respectively, of the variation in AMF community structure, verifying that they were the major factors shaping AMF community structure.

### AMF Community Co-occurrence Network

**FIGURE 3:**
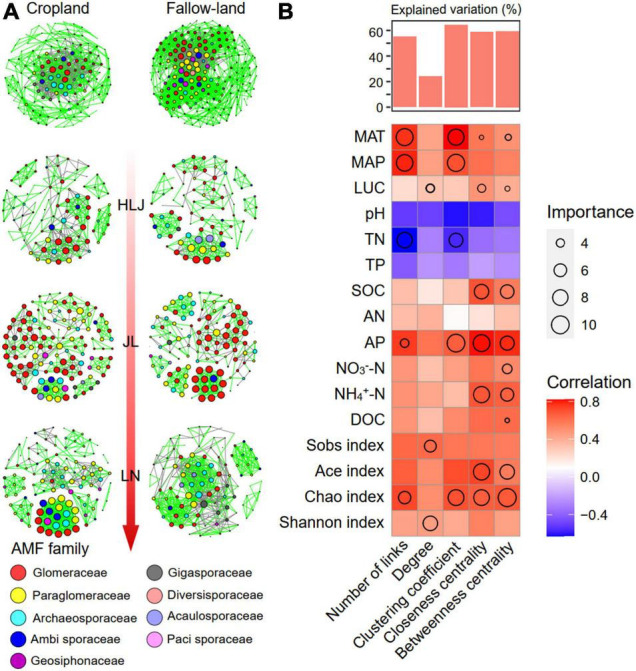
Topological features of co-occurrence network of the AMF community and their correlations with environmental variables. **(A)** Co-occurrence network analysis of the AMF community under different treatments. Each node represents an AMF OTU, and an edge represents a Spearman’s correlation with a correlation coefficient of > 0.8 or less than –0.8 that is statistically significant (FDR < 0.05); and **(B)** random forest regression estimating the effects of each environmental variable on network topological features. MAT, mean annual temperature; MAP, mean annual precipitation; DOC, dissolved organic carbon; SOC, soil organic carbon; AN, available nitrogen; AP, available phosphorus; TP, total phosphorus; TN, total nitrogen. NO_3_^–^-N, nitrate nitrogen; NH_4_^+^-N, ammonium nitrogen.

To determine the general effects of CC and LUC on associations among AMF species, eight networks were generated by combining all AMF taxa from three study sites and two land-use types (Figure 3A). Following this approach, we calculated topological features to characterize the changes in co-occurrence network patterns with CC and LUC treatments. The modularity values and average clustering coefficients of the empirical networks were higher than those of the random networks, indicating that all networks had a modular structure and “small-world” characteristics (Supplementary Table 6). All networks tended to be positive correlations rather than negative correlations, as positive links accounted for 67.38–83.47% of potential interactions. Multiple network topological metrics consistently indicated that the AMF co-occurrence network patterns varied greatly across the three study sites and different land-use types. The fallow-land assemblages formed larger networks with more nodes than the cropland network (Figure 3 and Supplementary Table 6). Fallow-land treatment contained more connections (links), a larger averaging degree and graph density than cropland, which indicates a more complex network for cropland treatment. The number of nodes, edges, average degree, clustering coefficients, and graph density from HLJ to LN showed different patterns of variation in the cropland and fallow-land treatments. The highest values of nodes, edges, clustering coefficients and graph densities were observed under fallow-land treatment in the LN communities, whereas the lowest values were observed in the HLJ community. However, under the cropland treatment, the maximum value of nodes, edges, average degree, and graph density occurred at the LN site. The proportion of positive edges gradually increased from HLJ site to LN site under the cropland treatment; however, under the fallow-land treatment, the proportion of positive edges was first decreased at JL site and then increased at LN site.

Next, we investigated the effects of environmental variables on topological features of AMF co-occurrence network by random forest modeling (Figure 3B). We found that different environmental factors contributed to the network complexity. For example, the climate condition and AMF diversity were the most important variables for predicting network complexity, including number of links, closeness centrality, and betweenness centrality. Other important variables for predicting network complexity were the AP, NO_3_^–^-N, and NH_4_^+^-N for closeness centrality and betweenness centrality, the TN and SOC for number of links.

### AMF Growth Parameters and GRSP Concentration

**FIGURE 4:**
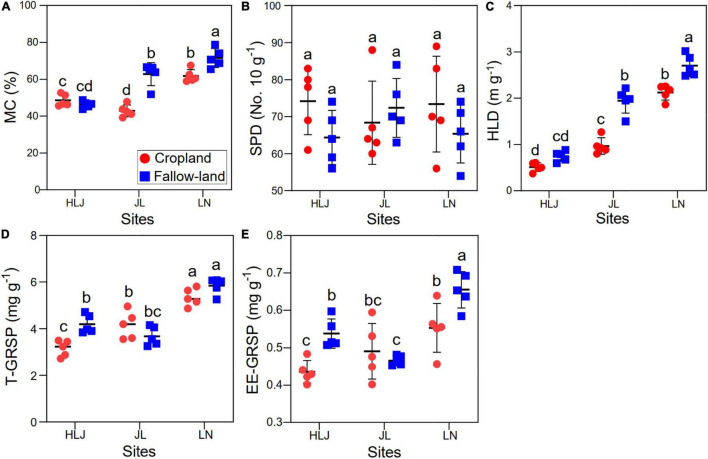
Effects of CC and LUC on the AMF growth parameters [**(A)** mycorrhizal colonization; **(B)** spore density; and **(C)** hyphal length density] and GRSP concentration [**(D)** T-GRSP concentration; and **(E)** EE-GRSP concentration]. Different letters indicate significant differences among the treatments (*p* < 0.05). CL = cropland; FL = fallow land.

Mycorrhizal colonization and hyphal length density were significantly affected by CC, LUC, and their interactions, but they had no effect on spore density (Figure 4 and Supplementary Table 4). Specifically, the mycorrhizal colonization and hyphal length density in the fallow land were higher compared with the cropland at JL and LN sites. Both CC and LUC had significant effects on T-GRSP and EE-GRSP (Figure 4 and Supplementary Table 4). There was no significant interactive effect of CC and LUC on EE-GRSP. T-GRSP and EE-GRSP were overall higher at LN site than at HLJ and JL sites, and the highest value was found in fallow land at LN site.

### Stability of Soil Aggregates

**FIGURE 5:**
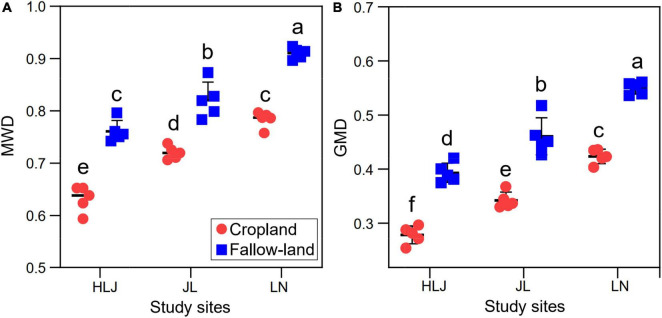
Effects of CC and LUC on MWD **(A)** and GMD **(B)**. Different letters indicate significant differences among treatments (*p* < 0.05).

Mean weight diameter and GWD are often used to quantify the stability of soil aggregates, and it is generally accepted that larger MWD and GWD are more stable for soil aggregates. Both CC and LUC had significant impacts on MWD and GMD (Figure 5 and Supplementary Table 4). There were no significant interactions between CC and LUC on MWD and GMD. Specifically, the MWD and GMD in fallow land were significantly higher than those of the cropland at all study sites, indicating that the fallow land improved the stability of soil aggregates.

### C Concentrations of T-GRSP and EE-GRSP

**FIGURE 6:**
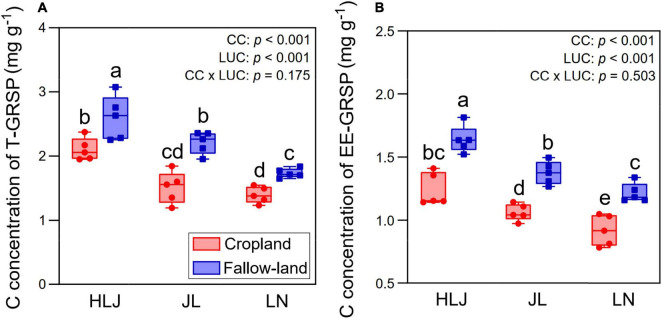
Effects of CC and LUC on C concentrations of T-GRSP **(A)** and EE-GRSP **(B)**. Means ± SD (*n* = 5) followed by different letters at the bar indicate significant differences (*p* < 0.05) among treatments.

Land-use conversion significantly increased the C concentrations of both T-GRSP and EE-GRSP (Figure 6). Compared to cropland, fallow land increased C concentration of T-GRSP by 24.2, 46.3, and 23.6%, while improved C concentration of EE-GRSP by 32.3, 29.5, and 32.0% at HLJ, JL, and LN sites, respectively. MAT was significantly and negatively correlated with C concentration of T-GRSP (*r* = 0.683, *p* < 0.001) and C concentration of EE-GRSP (*r* = 0.611, *p* < 0.001). Both CC and LUC had considerably effects on C concentrations of T-GRSP and EE-GRSP, but their interactive effect was not significant (*p* > 0.05).

### Aggregate-Associated SOC Concentration

**FIGURE 7:**
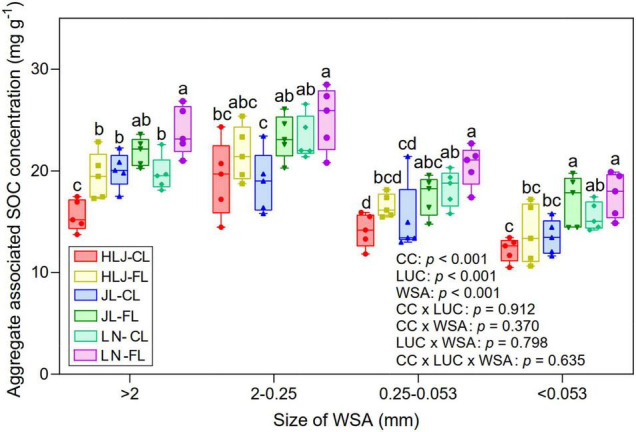
Aggregate-associated soil organic carbon (SOC) concentration (mg g^–1^) in different sizes of soil water stable aggregates under CC and LUC treatments. CL, cropland; FL, fallow land. The different letters above the error bars for the same aggregate fraction indicate significant differences at *p* < 0.05.

The SOC within different soil aggregate fractions was significantly impacted by CC at all study sites (*p* < 0.001, Figure 7). Among the aggregate fractions, small macro-aggregate (2–0.25 mm) fractions sequestrated more C, followed by large macro-aggregates (>2 mm). The cropland at LN study site had a significantly lower SOC concentration within >2-mm fraction than other study sites. However, for the 2–0.5-mm fractions, LUC did not influence the aggregate-associated SOC concentration at HLJ and LN study sites. The aggregate-associated SOC concentration increased with the increase of MAT for all different soil aggregate fractions. There were significant effects of CC, LUC, or aggregate size fraction on aggregate-associated SOC concentration (*p* < 0.001), but their interactive effects were not significant (*p* > 0.05).

## Discussion

Exploring the responses of microbial communities and their effects on environmental changes is crucial to gaining a better understanding of the mechanisms that maintain the functions of terrestrial ecosystems. Here, this study elucidated the mechanisms underpinning the response of AMF community composition, diversity, and co-occurrence networks to CC and LUC and uncovered the roles of AMF in maintaining soil aggregate stability and C sequestration in an 8-year experiment in relatively high-latitude regions. Our study highlighted that CC and LUC exerted strong effects on MWD and GMD. We also observed the major role of AMF diversity, network complexity, and GRSP in the soil aggregate stability and carbon sequestration.

Changes in the structure and diversity of AMF community are closely linked to their ecosystem function (e.g., soil aggregation and carbon sequestration). Therefore, revealing the effects of environmental variables on AMF communities can further enhance our understanding of how ecosystem processes respond to environmental changes. The results showed that eight genera were detected in the soil samples, whereas *Glomus*, *Paraglomus*, and *Archaeospora* were the three most abundant genera at all study sites, suggesting that they had a wide ecological niche and strong adaptability in both natural and managed environments (Öpik et al., 2013). The genus *Glomus* could be widely detected in agricultural fields, indicating that it was better adapted to disturbed environments, which could be attributed to its higher sporulation rates and good symbiotic relationships with plant roots (Yang et al., 2015). AMF species belonging to *Paraglomus* could be distributed in various types of soil and live in almost all climate zones worldwide (Gosling et al., 2014). The genus *Acaulospora* has been considered as a stress-tolerant genus because it is frequently detected under harsh climatic conditions in acidic or alkaline soils as well as heavily contaminated conditions, suggesting its ability to withstand different environmental conditions and to adapt to various ecosystems (Yang et al., 2015). In contrast, a very low relative abundance of *Diversispora* was observed among all AMF sequences, which was consistent with the findings of Zhang et al. (2020) and implied the contrasting habitat preferences between abundant and rare AMF genera in agricultural ecosystems. In addition, the widespread distribution of the dominant genera indicated their potential contributions to the functions of agricultural ecosystem. Therefore, the different abundance patterns among AMF genera could help us to screen the functional AMF species in the future studies.

The α-diversity of the AMF community was significantly influenced by both CC and LUC, and it was more sensitive to LUC than CC (Figure 1B). This study showed that the richness of the AMF community increased with temperature, according to the metabolic theory of ecology (Brown et al., 2004). Temperature has been considered one of the most profound drivers of ecological community structure and could provide a powerful framework for predicting the effects of CC on biodiversity (Zhou et al., 2016). Based on the results of the random forest model, the α-diversity of the soil AMF community was well predicted by increased temperature in this study (Supplementary Figure 3), which could support the metabolic theory of ecology proposed by Brown et al. (2004). Compared with fallow land where plants grew freely, cropland provided a rather unique habitat because of the disturbing conditions generated by agricultural management activities. A previous study showed that biotic and abiotic disturbances increased the richness of the bacterial community in agricultural fields (Jiao et al., 2019). However, the richness and α-diversity of the AMF community did not increase in cropland, and we observed that the richness and α-diversity were higher in fallow land than in cropland (Figure 1B). This finding could be reasonably explained by the observation that cropland inhibited the growth of AMF and therefore decreased the species turnover rate in disturbed environments (Figure 4). On the other hand, the larger number of plant species in fallow land than cropland could create a more heterogeneous environment and consequently increase the α-diversity of AMF by reducing the niche competition and overlap based on niche theory. Therefore, the results highlight the importance of AMF to maintain agricultural ecosystem functions as indicated by Benkwitt et al. (2020) who found positive relationships between biodiversity and ecosystem functioning (BEF).

In natural environments, soil microorganisms generally tend to co-occur and to form a complex network with positive, negative, and neutral relationships (Faust and Raes, 2012), which may have a stronger influence on community structure than environmental variables (de Menezes et al., 2014). CC and LUC could influence not only AMF community structure but also AMF co-occurrence relationships. The positive interactions could be associated with cross-feeding and niche overlap within the community, whereas the negative associations were attributed to competition and amensalism (Faust and Raes, 2012). In this study, the number of positive links in all networks was greater than the number of negative links, indicating more AMF cooperation than competition. This phenomenon was probably attributed to the facilitative interactions among AMF taxa developed in a longer period of coevolution processes (Zhang et al., 2014). Moreover, the variations in the AMF interactions of agricultural soils in response to cropland and fallow land were explored through co-occurrence networks. The results showed that cropland decreased the number of network connections, indicating that more convergent AMF communities were generated in cropland. It is widely accepted that co-occurring species generally share similar ecological niches or life-history strategies and that they are often more adaptable to homogeneous habitats (Jiao et al., 2019). The agricultural practice probably reduced the homogeneity of the soil, resulting in a decrease in the coexistence of AMF species and a reduction in the convergence of AMF taxa. In addition, both modularity and clustering coefficient were significantly higher than the corresponding random networks, indicating that AMF co-occurrence networks had a modular structure and typical small-world features (Watts and Strogatz, 1998; de Celis et al., 2022). Modularity reflects synergistic/competitive relationships, generating non-random patterns in network structure and ultimately increasing the complexity of microbial networks. Modularity can be considered a strategy to maintain the stability of the AMF community, resulting in faster and better adaptation of AMF species to environmental changes (Krause et al., 2003). The higher AMF diversity and more complex networks were detected in fallow land compared with cropland in this study (Figure 1B and Supplementary Table 6). This result was consistent with the previous theory that more microbial taxa imply more potential interactions. The increase in complexity of the AMF network from cropland to fallow land may be attributed to the increase in plant diversity, which was regarded as a selective force for soil microbiome assembly and could increase the chance for coevolution (Xu et al., 2022). Moreover, Glomeraceae, Paraglomeraceae, and Archaeosporaceae had the higher values of topological features and were more often located in central ecological positions than other families, indicating their potential roles in maintenance of ecosystem multifunctionality. Both MAP and MAT were the important factors in regulating the number of links and clustering coefficients, whereas MAT was remarkably correlated with closeness centrality and betweenness centrality (Figure 3). Based on the metabolic theory of ecology, it is predicted that the increased temperature stimulates various biological interactions (symbiosis, parasitism, predation, and competition) due to more active individual metabolic processes and faster growth at higher temperatures (Brown et al., 2004). The positive effects of MAP on the complexity of AMF networks may be attributed to the various sensitivities of AMF taxa to soil nutrients and moisture caused by precipitation (de Vries et al., 2018; Wang et al., 2021). However, CC (MAT and MAP) exhibited different effects on co-occurrence relationships among AMF taxa and were not correlated with the degrees and centrality (Figure 3). This contrary observation implied the different habitat preferences of AMF taxa in fallow land and cropland.

Some studies have shown that aggregates are essential for nutrient storage, organic matter turnover, and water infiltration, and their stability has been regarded as an indicator of soil quality (Gelaw et al., 2015). MWD and GMD are frequently utilized to quantify the stability of soil aggregates, and it is accepted that larger MWD and GWD values indicate higher stability of soil aggregates (Liu et al., 2021). The MWD and GMD values were significantly higher in fallow land than in cropland, indicating that the soil structure was more sensitive to agricultural activities. In addition, the MWD and GMD at LN site were higher than those at HLJ and JL sites, suggesting that CC may have a positive impact on the stability of soil aggregates, whereas cropland reduced the MWD and GMD probably because the agricultural practice inhibited the growth and diversity of AMF (Figures 1B, 4). Our results were consistent with previous studies (Qian et al., 2018), which revealed that soil aggregates from abandoned lands had greater stability than those from cultivated lands. The reduced/no-tillage practices could decrease soil perturbation, thereby improving soil infiltration and promoting the formation of a stable soil structure (Sithole et al., 2019). In addition, the changes in the microbial community could also influence soil aggregate stability (Zhao et al., 2018). It has been reported that fungi display more impressive functions than bacteria in stabilizing aggregates (Leifheit et al., 2014). The ubiquitous soil AMF community could directly contribute to the stability of soil aggregates by extraradical fungal hyphae (Rillig and Mummey, 2006; Peng et al., 2013) or indirectly contribute by altering the morphological and biochemical characteristics of host plants (Borie et al., 2008). Our research indicates that CC and LUC could regulate GRSP and soil aggregate stability mainly by regulating AMF diversity, network complexity, and growth parameters based on the results of random forest model (Supplementary Figure 4). Importantly, GRSP was located in the core position to influence the stability of soil aggregates and was the most important factor for explaining soil aggregation (Li et al., 2022). Therefore, changes in GRSP in the aggregate fraction may have a direct impact on aggregate stability. The results of this study showed that both EE-GRSP and T-GRSP were positively correlated MWD and GMD (Supplementary Figure 5), which was consistent with most previous studies (Wu et al., 2012; Li et al., 2022). However, the effect of GRSP on soil aggregate stability was greatly linked to the soil particle size. Xiao et al. (2020) discovered that the T-GRSP concentration in < 0.25 mm aggregates and DE-GRSP concentration in < 0.25 mm aggregates were linearly correlated with MWD, indicating that GRSP was mainly related to soil micro-aggregate stability. Hence, this study expands our understanding of the effects of CC and LUC on soil health and function and highlights the dominant role of AMF and the GRSP secreted by AMF in the stability and maintenance of soil structure in agricultural ecosystems.

**FIGURE 8:**
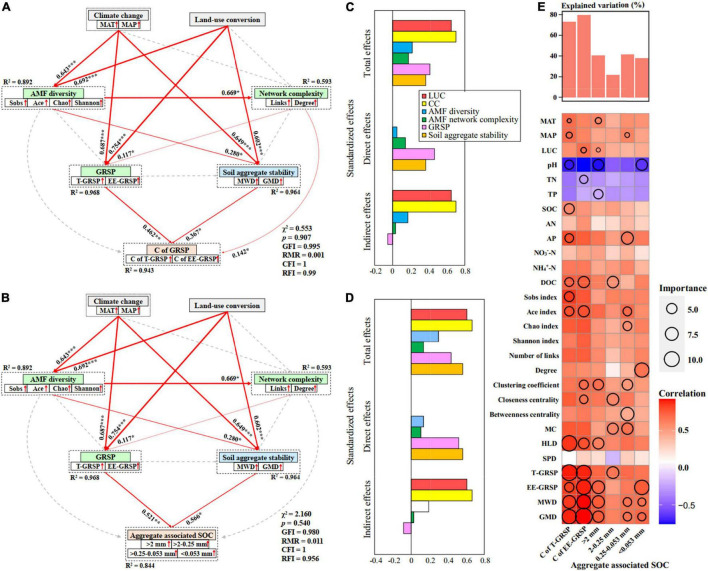
Structural equation model analysis (SEM) that examining the effects of CC and LUC on the C concentration of GRSP **(A)** and soil aggregate-associated SOC concentration **(B)** *via* pathways of AMF diversity, AMF network complexity, GRSP and soil aggregate stability in cropland and fallow land. Red arrows indicate positive relationships. Continuous arrows and dashed arrows indicate significant relationships and non-significant relationships, respectively. The arrow width is proportional to the strength of the relationship. Double-layer rectangles represent the first component from the PCA conducted for AMF diversity, AMF network complexity, GRSP and soil aggregate stability. The red symbol “↑” indicates a positive relationship between the variables and the first component from the PCA. The numbers adjacent to the arrows are standardized path coefficients, which reflect the effect size of the relationship. The proportion of variance explained (*R*^2^) appears alongside each response variable in the model. The final models yielded good fits to the data; Standardized effects of CC, LUC, AMF diversity, network complexity, GRSP, and soil aggregate stability on the C concentration of GRSP **(C)** and soil aggregate-associated SOC concentration **(D)**; and **(E)** random forest model estimating the effects of each environmental variable on the C concentration of GRSP and soil aggregate-associated SOC concentration.

Previous study has reported that SOC concentration was higher in no-till than the conventional-till treatment (Sekaran et al., 2021). Our results showed that fallow land exhibited an increase in SOC concentration compared to the cropland treatment. This is mainly because cropland can enhance soil microbial respiration and improve the decomposition rate of SOC through their destructive action. On the contrary, less soil disturbance and stronger soil aggregate stability in fallow land (Figure 5) promoted the protection of SOC within soil aggregates. This study also found significantly positive correlations between SOC with weight percentage of large macro-aggregates (*r* = 0.454, *p* = 0.012) and small macro-aggregates (*r* = 0.486, *p* = 0.006). There was the highest SOC concentration (10.96 mg g^–1^) within the macro-aggregates (>2 mm and 2–0.25 mm) under fallow land at HLJ study site (Figure 7). This study further suggested that CC and LUC exhibited strongest total effects and indirect effects on C of GRSP and soil aggregate-associated SOC through regulating AMF diversity, network complexity, GRSP, and stability of soil aggregates based on both structural equation model and random forest regression (Figure 8). The increase in aggregate-associated SOC concentration under fallow land was mainly attributed to the large C input because of high plant residue retention and minimum soil disturbance (Lange et al., 2015). Moreover, soil aggregation is greatly linked to soil aggregate-associated SOC because macro-aggregates showed beneficial effect on the preservation of SOC and C sequestration (Abrar et al., 2020). Herein, LUC from cropland to fallow land can improve SOC sequestration by enhancing aggregate-associated SOC and stability of soil aggregates, which can be considered as a promising strategy contributed to the mitigation of climate change (Burdukovskii et al., 2019).

## Conclusion

Our study provides evidence that the effects of CC and LUC on soil aggregate stability, C-GRSP, and aggregate-associated SOC were regulated by AMF diversity, network complexity, and their secreted GRSP. The results suggested the potential role of AMF in soil C sequestration and climate change mitigation. The long-term maintenance of fallow-land system effectively promoted the AMF diversity and network complexity, GRSP, C-GRSP, and soil aggregate-associated SOC. Moreover, the water-stable aggregates and their stability (MWD and GMD) were also increased under long-term fallow-land treatment. The results indicated that the fallow land enhanced the C storage capacity of the soil aggregates. Both random forest regression and structural equation model suggested that CC and LUC had strong effects on C-GRSP and soil aggregate fractions through regulating diversity and network complexity of AMF community, and the GRSP secreted by AMF. Our study emphasized the beneficial effects of AMF diversity and network complexity on soil aggregation and C sequestration in soils and highlights the role of AMF in mediating the effects of CC and LUC on soil health and C sequestration.

## Data Availability Statement

The datasets presented in this study can be found in online repositories. The names of the repository/repositories and accession number(s) can be found in the article/Supplementary Material.

## Author Contributions

YY, XW, and DW designed and conceived the experiment. YY, WL, JX, and PG carried out the experiments and collected the empirical data. YY, LC, XW, and DW performed the data analysis. YY and WL wrote the manuscript with contributions from XW and DW. All authors read and approved the final manuscript.

## Conflict of Interest

The authors declare that the research was conducted in the absence of any commercial or financial relationships that could be construed as a potential conflict of interest.

## Publisher’s Note

All claims expressed in this article are solely those of the authors and do not necessarily represent those of their affiliated organizations, or those of the publisher, the editors and the reviewers. Any product that may be evaluated in this article, or claim that may be made by its manufacturer, is not guaranteed or endorsed by the publisher.
