# Supplementary material for: Fallow Land Enhances Carbon Sequestration in Glomalin and Soil Aggregates Through Regulating Diversity and Network Complexity of Arbuscular Mycorrhizal Fungi Under Climate Change in Relatively High-Latitude Regions
Source: Front Microbiol. 2022 Jul 4;13:930622. doi: 10.3389/fmicb.2022.930622 (PMC9292920; doi:10.3389/fmicb.2022.930622)
Supplement: Supplementary file 1 [file Data_Sheet_1.docx]

**SUPPORTING MATERIAL**

**Fallow-land enhances carbon sequestration in glomalin and soil aggregates through regulating diversity and network complexity of arbuscular mycorrhizal fungi under climate change in relatively high-latitude regions**

Yurong Yang^1,2#^, Wenbo Luo^2#^, Jiazheng Xu^1^, Pingting Guan^2^, Liang Chang^3^, Xuefeng Wu^1*^, Donghui Wu^2,3^*

^1^ *Key Laboratory of Vegetation Ecology, Ministry of Education,* *Northeast Normal University, Changchun 130024, China*

^2^ *State Environmental Protection Key Laboratory of Wetland Ecology and Vegetation Restoration, Northeast Normal University, Changchun 130117, China*

^3^ *Key Laboratory of Wetland Ecology and Environment, Northeast Institute of Geography and Agroecology, Chinese Academy of Sciences, Changchun 130102, China*

***Correspondence** **authors**

Xuefeng Wu (XW)

Tel: +86 18443173890; Fax: +86 0431-85099590; E-mail: wuxf112@nenu.edu.cn

Donghui Wu (DW)

Tel: +86 15904308293; Fax: +86 0431-89165610; E-mail: [wudonghui@iga.ac.cn](mailto:wudonghui@iga.ac.cn)

^#^These authors contributed to the work equally and should be regarded as co-first authors.

# Method 1: Distribution and Stability of Water-Stable Aggregation (WSA)

Soil aggregate distribution and water-stable aggregation (WSA) were assessed using a wet-sieving apparatus (Karlen et al., 2013). Briefly, 100 g of dried soil was transferred to a set of three stacking sieves with openings of 2 mm, 0.25 mm, and 0.053 mm, resulting in the collection of four aggregate size classes: >2 mm, 2-0.25 mm, 0.25-0.053 mm, and <0.053 mm. The sieves were immersed in distilled water and agitated for 10 min with a vertical stroke of 1.3 cm and a speed of 30 cycles min^-1^. The soil samples were oven-dried (105 °C) and weighed to determine the soil aggregate size distribution. The mean weight diameter (MWD) and geometric mean diameter (GMD) were used to represent the soil aggregate stability (Lal and Shukla, 2004):

$$\mathrm{MWD}=\sum_{i=1}^{n} (Y_{i}\times X_{i})$$

$$GMD=exp(\sum_{i=1}^{n} W_{i}\times lnX_{i}/\sum_{i=1}^{n} W_{i})$$

where Y_i_ is the proportion of each sieve from the whole sample weight, X_i_ is the mean diameter of the size class (mm), W_i_ is the weight of the aggregates of each size class (g) and n is the total number of sieves utilized for separation.

# Method 2: AMF Growth Parameters

Plant roots were washed with tap water, cleared in 10% KOH, bleached in H_2_O_2_, acidiﬁed with 1% lactic acid, and then stained with 0.05% trypan blue (Phillips and Hayman, 1970). The mycorrhizal colonization (MC) was determined according to Biermann and Linderman (1981) by monitoring the proportion of root length colonized by AMF. AMF spores were extracted from 20 g of soil using the wet-sieving and decanting method (Gerdemann and Nicolson, 1963), and the number of spores was counted under a dissecting microscope. The spore density (SPD) was expressed as the number of spores per 10 g dried soil (No. 10 g^-1^). AMF hyphae in soil samples were isolated on a 38 μm sieve, resuspended and stained with a 0.05% (w/v) trypan blue solution. The hyphal length density (HLD) of AMF was estimated under a microscope based on the grid-line intersection method (Jakobsen et al., 1992).

# Method 3: DNA Extraction, Pyrosequencing and Data Processing

The total microbial genomic DNA in the soil samples was extracted from 0.4 g of frozen soil by a PowerSoil DNA extraction kit (MoBio, Carlsbad, USA) according to the manufacturer’s recommendations. The final DNA concentration and purification were detected with a NanoDrop 2000 UV–vis spectrophotometer (Thermo Scientific, Wilmington, USA), and the DNA quality was evaluated by 1.5% agarose gel electrophoresis. Nested PCR was carried out to amplify the partial small subunit (SSU) region of the 18S rRNA gene of AMF. The AML1 and AML2 primer set (AML1, 5’-ATCAACTTTCGATGGTAGGATAGA-3’; AML2, 5’-GAACCCAAACACTTTGGTTTCC-3’) was employed in the first round of PCR amplification, while the second round of PCR amplification was performed using the AMV4.5NF and AMDGR primer set (AMV4.5NF, 5’-AAGCTCGTAGTTGAATTTCG-3’; AMDGR, 5’-CCCAACTATCCCTATTAATCAT-3’) based on Ji et al. (2021).

The purified, barcode-tagged amplicons from each cDNA library were merged into equimolar concentrations and paired-end sequenced (2 × 300) on an Illumina MiSeq PE300 platform (Illumina, USA) by Majorbio Co., Ltd. (Shanghai, China) (Caporaso et al., 2012). The Illumina sequencing raw read data deposited in the Sequence Read Archive (SRA) are available in the NCBI SRA portal with PRJNA797899, bioproject ID. More details about the PCR conditions and quality assessment are provided in Supplementary Material Table S1.

The raw sequences were filtered for quality trimming and removal of adapters from FASTQ data to obtain qualified reads by Trimmomatic, and the resulting sequences were merged using FLASH (Magoc and Salzberg, 2011). The short sequences, singleton sequences and noisy reads were removed based on the denoising protocol using the USEARCH algorithm (Edgar, 2010). The quality reads were clustered into operational taxonomic units (OTUs) with a similarity threshold of 97% by UPARSE (Edgar, 2010). Taxonomic assignment of the representative sequence from each OTU was performed using the MaarjAM database. Prior to statistical analysis, the sequence number in the different samples is randomly resampled until the smallest sampling size has been reached, thereby assuring a uniform sequence depth.

## **Co-occurrence Network Analysis**

Co-occurrence network analysis has been increasingly performed in microbial ecology in recent years to better understand microbial community structure and to characterize potential intracommunity interactions among species. Eight co-occurrence networks of the soil AMF communities from cropland and fallow-land at three study sites were constructed based on OTU relative abundances. We calculated the Spearman correlation coefficient in the soil AMF community in R with the package Hmisc (Harrell and Frank, 2008). Only the robust correlations with |r| ≥ 0.6 and p < 0.01 were kept to construct networks. Thereafter, multiple p-values were adjusted according to the false discovery rate (FDR) with the Benjamini controlling procedure (Benjamini et al., 2006). The co-occurrence networks were visualized, and the topological features (i.e., numbers of nodes and edges, modularity, network diameter, clustering coefﬁcient, graph density and average path length) were estimated by the igraph R package (Csárdi and Nepusz, 2006). The modular structure of the microbial community was evaluated via the modularity index (Lambiotte et al., 2015). The nodes with a high degree (top 1% of interactions) were regarded as possible keystone species in the networks (Banerjee et al., 2018). Moreover, 1,000 Erdös-Rényi random networks with the same number of nodes and edges as the real networks were generated for comparison with the real networks by the igraph R package (Erdös and Rényi, 1960).

# REFERENCES

Banerjee, S., Schlaeppi, K., and van der Heijden, M. G. A. (2018). Keystone taxa as drivers of microbiome structure and functioning. *Nat. Rev. Microbiol.* 16, 567-576.

Benjamini, Y., Krieger, A. M., and Yekutieli, D. (2006). Adaptive linear step-up procedures that control the false discovery rate. *Biometrika* 93, 491-507.

Biermann, B., and Linderman, R. G. (1981). Quantifying vesicular-arbuscular mycorrhizae: A proposed method towards standardization. *New Phytol.* 87, 63-67.

Caporaso, J. G., Lauber, C. L., Walters, W. A., Berg-Lyons. D., Huntley, J., Fierer N, et al. (2012). Ultra-high-throughput microbial community analysis on the Illumina HiSeq and MiSeq platforms. *ISME J.* 6, 1621-1624.

Csárdi, G., and Nepusz, T. (2006). The igraph software package for complex network research. *Interjournal ComplexSyst.* 1695, 1-9.

Edgar, R. C. (2010). Search and clustering orders of magnitude faster than BLAST. *Bioinformatics* 26, 2460-2461.

Erdös, P., and Rényi, A. (1960). On the evolution of random graphs. *Publ. Math. Inst. Hung. Acad. Sci.* 5, 17-60.

Gerdemann, J. W., and Nicolson, T. H. (1963). Spores of mycorrhizal *Endogone* species extracted from soil by wet sieving and decanting. *Trans. Br. Mycol. Soc.* 46, 235-244.

Harrell, J., and Frank, E. (2008). Hmisc: Harrell miscellaneous. *R Package Version* 3.5-2.

Jakobsen, I., Abbott, L. K., and Robson, A. D. (1992). External hyphae of vesicular-arbuscular mycorrhizal fungi associated with *Trifolium subterraneum* L. *New Phytol.* 120, 371-380.

Ji, L., Yang, Y., Yang, N., Khan, A., and Yang, L. (2021). Seasonal variation of diversity and co-occurrence patterns of arbuscular mycorrhizal fungal communities in mixed broadleaf-conifer forests. *Appl. Soil Ecol.* 158, 103782.

Karlen, D. L., Cambardella, C. A., Kovar, J. L., and Colvin, T. S. (2013). Soil quality response to long-term tillage and crop rotation practices. *Soil Tillage Res.* 133, 54-64.

Lal, R., and Shukla, M. J. (2004). *Principles of Soil Physics*. Marcel Dekker, New York.

Lambiotte, R., Delvenne, J. C., and Barahona, M. (2015). Random walks, Markov processes and the multiscale modular organization of complex networks. *IEEE Trans. Netw. Sci. Eng.* 1, 76-90.

Magoc, T., and Salzberg, S. L. (2011). FLASH: fast length adjustment of short reads to improve genome assemblies. *Bioinformatics* 27, 2957-2963.

Phillips, J. M., Hayman, D. S. (1970). Improved procedures for clearing roots and staining parasitic and vesicular-arbuscular mycorrhizal fungi for rapid assessment of infection. *T. Brit. Mycol. Soc.* 55, 158-161.


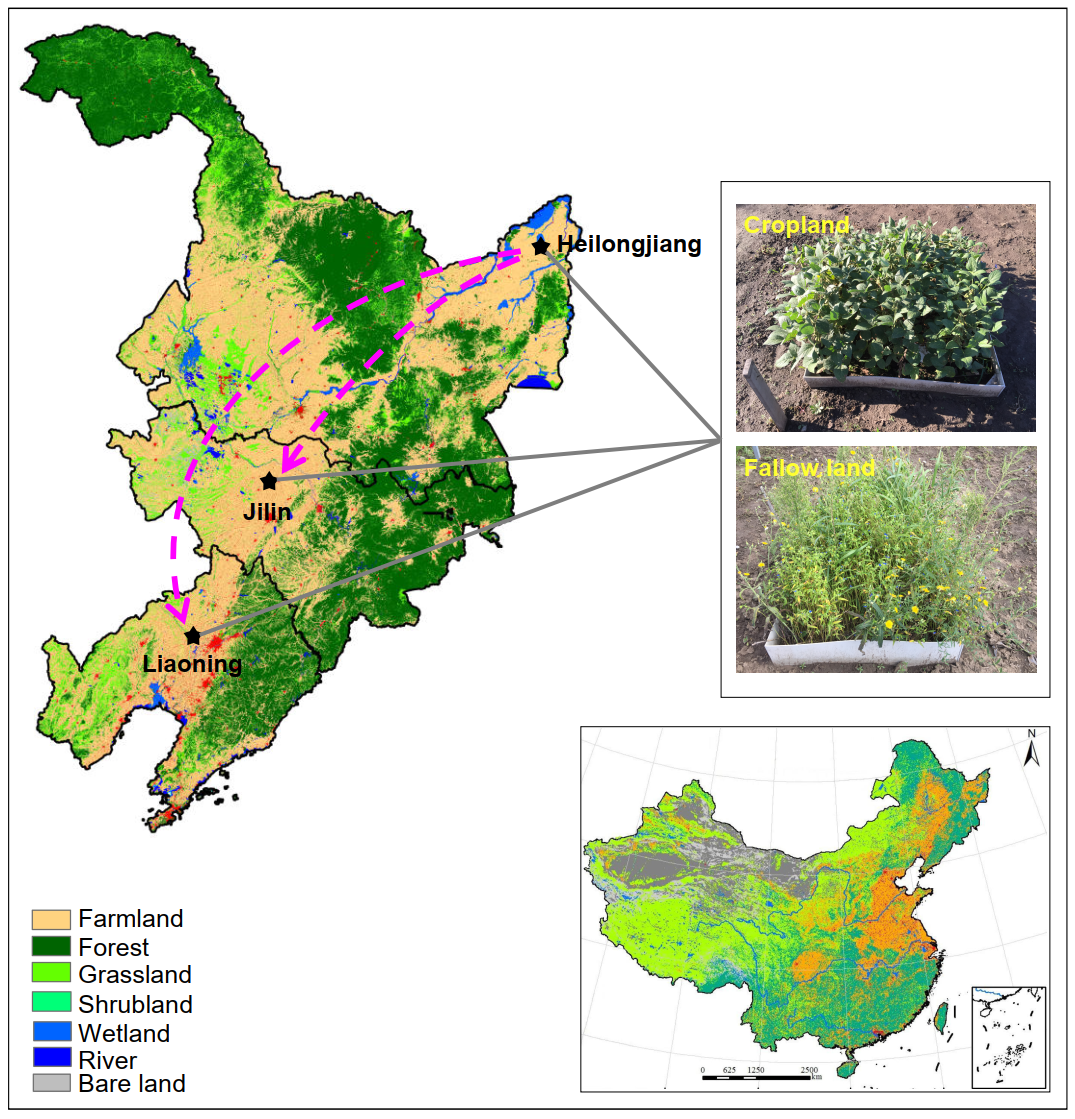


**Supplementary Figure 1.** Design of soil plots transplantation experiment. Totally, 30 plots from soybean fields with a 30-year cropping history at Heilongjiang site were transplanted to three study sites (Heilongjiang, HLJ; Jilin, JL; and Liaoning, LN) with distinct climate conditions to simulate climate change and land-use conversion from cropland to fallow-land in mid- to high-latitude regions.


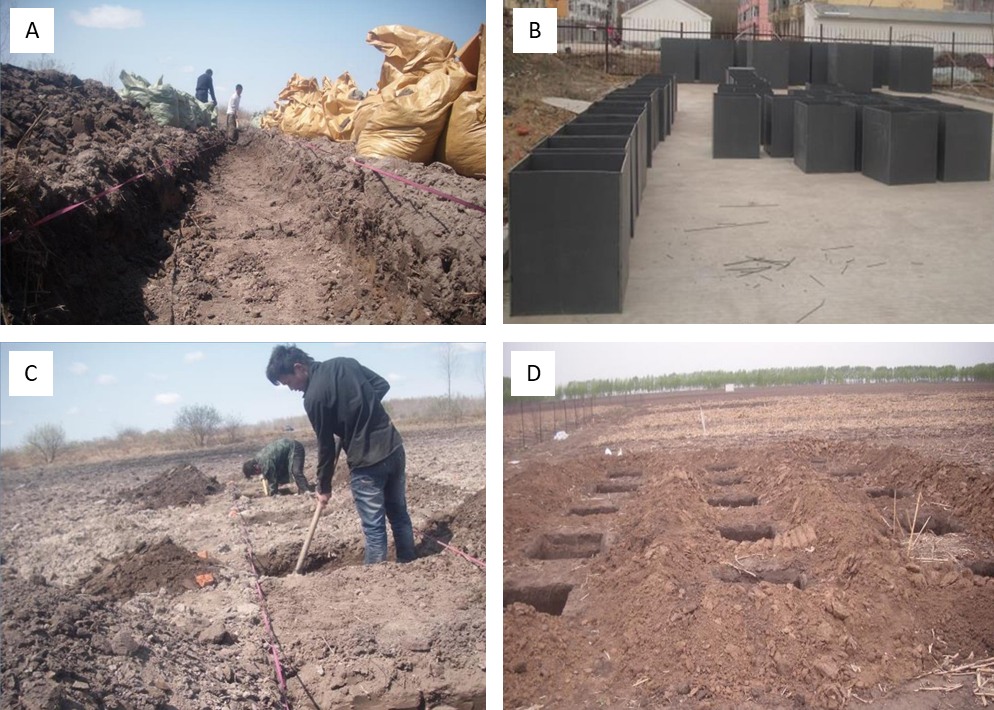


**Supplementary Figure 2.** The experimental setting showing that: A, the soil plots were excavated in five layers (20 cm per layer); B, a view of the PVC boxes (1 m in length, 1 m in width and 1 m in depth) where the soil plots were restacked; C and D, a view of the right size pits for PVC boxes.


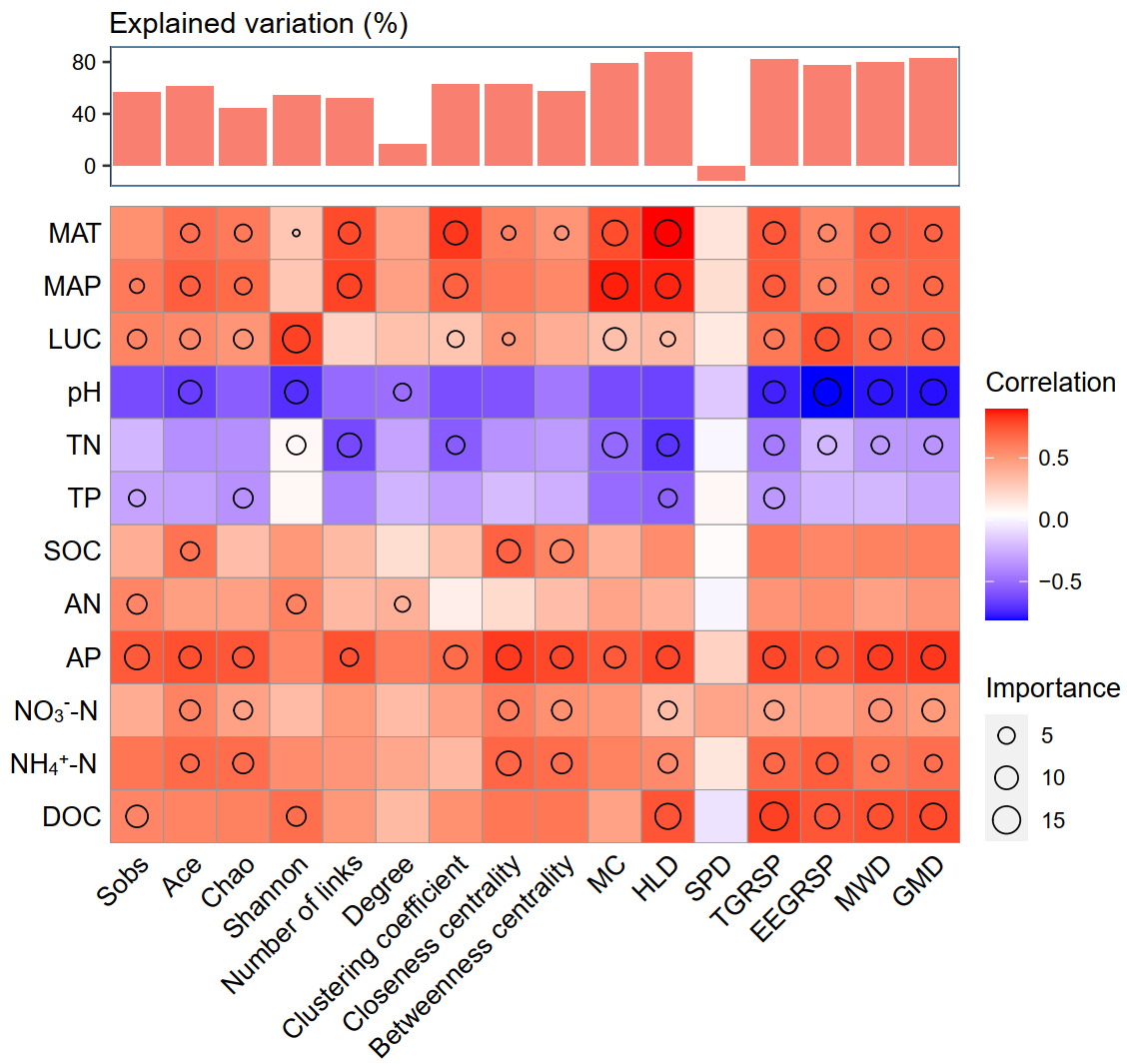


**Supplementary Figure 3.** Random forest model estimating the effects of environmental variables on AMF α-diversity, network complexity, growth parameters, GRSP concentration and soil aggregate stability. MAT = mean annual temperature; MAP = mean annual precipitation; SOC = soil organic carbon; AN = available nitrogen; AP = available phosphorus; TP = total phosphorus; TN = total nitrogen; NO_3_^-^-N = nitrate nitrogen; NH_4_^+^-N = ammonium nitrogen; MC = mycorrhizal colonization; HLD = hyphal length density; SPD = spore density.


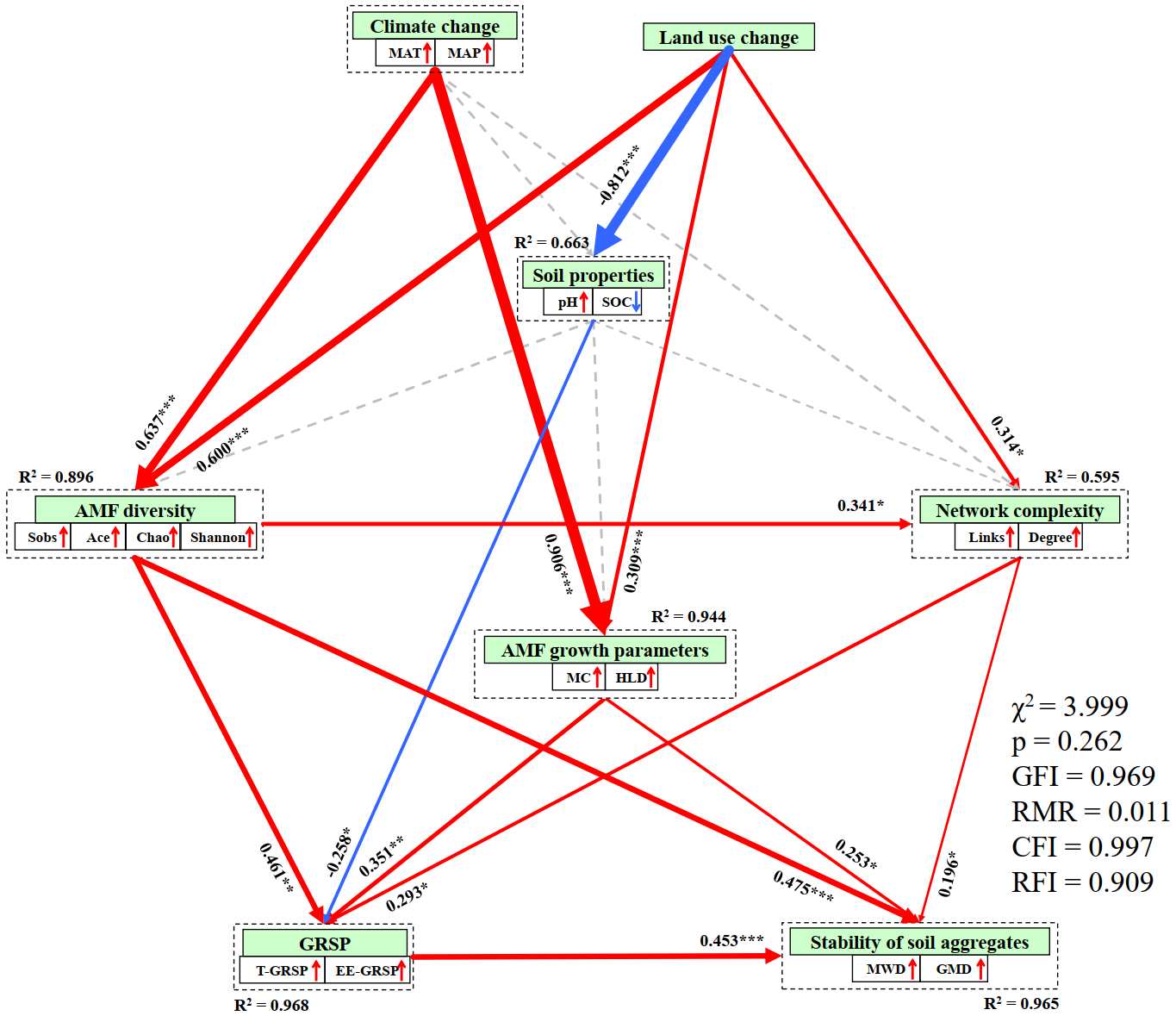


**Supplementary Figure 4.** Structural equation model analysis (SEM) that examining the effects of CC and LUC on the soil aggregate stability via pathways of soil properties, AMF diversity, AMF network complexity, AMF growth parameters, and GRSP in cropland and fallow-land. Red arrows and blue arrows indicate positive and negative relationships, respectively. Continuous arrows and dashed arrows indicate significant relationships and non-significant relationships, respectively. The arrow width is proportional to the strength of the relationship. Double-layer rectangles represent the first component from the PCA conducted for soil properties, AMF diversity, AMF network complexity, AMF growth parameters, and GRSP. The red symbol ‘↑’ and blue symbol ‘↓’ indicate a positive and negative relationship between the variables and the first component from the PCA, respectively. The numbers adjacent to the arrows are standardized path coefficients, which reflect the effect size of the relationship. The proportion of variance explained (R^2^) appears alongside each response variable in the model. The final models yielded good fits to the data.


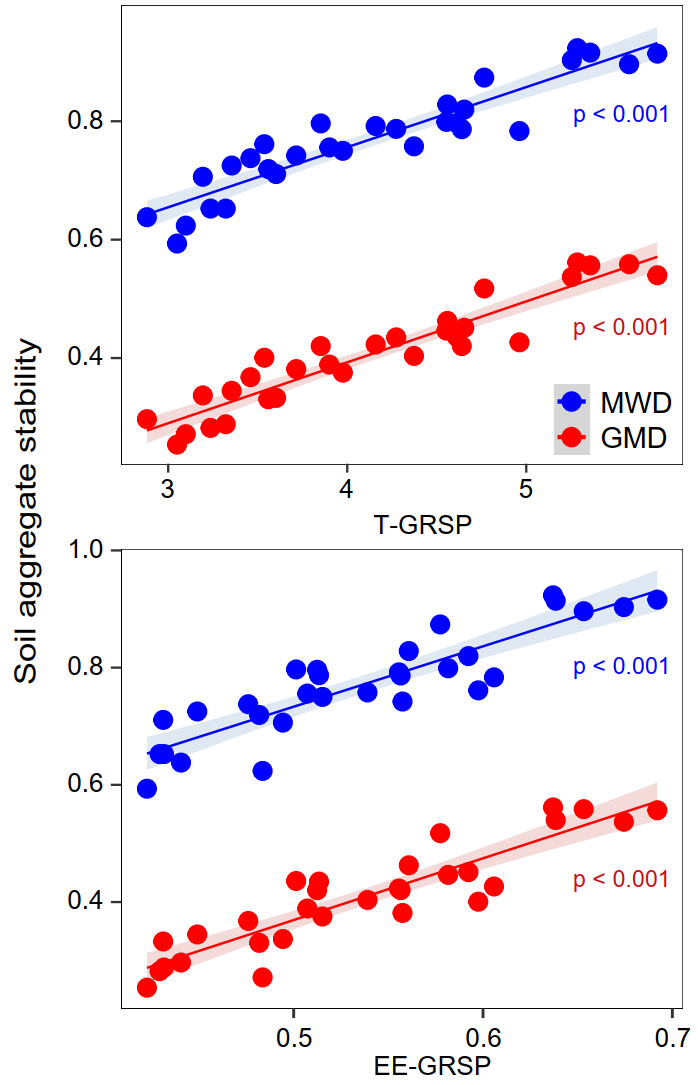


**Supplementary Figure 5.** Relationship between GRSP (T-GRSP and EE-GRSP) and soil aggregate stability (MWD and GMD).

**Supplementary Table 1.** The geographical location and basic climate parameters of the three stations.

| Station | Abbreviation | Latitude  (^o^) | Longitude  (^o^) | Altitude  (m) | MAT  (℃) | MAP  (cm) |
| --- | --- | --- | --- | --- | --- | --- |
| Sanjiang Field Experimental Station | HLJ | 47.58 | 133.52 | 66.4 | 3.3 | 579.9 |
| Changchun Agricultural Experimental Station | JL | 44.20 | 125.55 | 236.8 | 6.4 | 592.9 |
| Liaozhong Agricultural Experimental Station | LN | 41.68 | 122.78 | 30.7 | 8.5 | 651.2 |

MAT, mean annual temperature; MAP, mean annual precipitation.

**Supplementary Table 2.** Primer sets and thermal profiles used in PCR amplification.

|  | Primer | Sequence (5′-3′) | PCR conditions |
| --- | --- | --- | --- |
| AMF  SSU  rRNA | AML1 | ATCAACTTTCGATGGTAGGATAGA | 3 min at 95°C followed by 32 cycles of 30 s at 95°C,30 s at 55°C, 45 s at 72°C, and 10 min at 72°C for the last cycle |
|  | AML2 | GAACCCAAACACTTTGGTTTCC |  |
|  | AMV4.5NF | AAGCTCGTAGTTGAATTTCG | 3 min at 94°C followed by 30 cycles of 30 s at 95°C, 30 s at 55°C, 45 s at 72°C, and 10 min at 72°C for the last cycle |
|  | AMDGR | CCCAACTATCCCTATTAATCAT |  |
| Reaction system | Both PCR reactions were performed in triplicate in a total volume of 20 μL, containing 4 μL of 5 x FastPfu Buffer, 2 μL of 2.5 mM dNTPs, 0.4 μL of each primer (10 μM), 0.4 μ L of FastPfu Polymerase, 1 μL of template DNA (approximately 10 ng), 0.2 μL of BSA, and 10.8 μL of dd H_2_O. An 8 bp sequence barcode was added as a tag to distinguish the PCR products from one another. | | |

**Supplementary Table 3.** The explanatory power of the principal components analysis (PCA) results for each group (climate change, soil properties, AMF diversity, AMF growth parameters, GRSP and stability of soil aggregates). Value means the correlation coefficient between the variable and scores for rows in the main matrix (PC1).

| Variables | PC1 | Variables | PC1 |
| --- | --- | --- | --- |
| **Climate change** | | **Soil properties** | |
| MAT | 0.95*** | pH | 0.81*** |
| MAP | 0.95*** | SOC | 0.81*** |
| Cumulative (%) | 94.75 | Cumulative (%) | 80.75 |
| **AMF diversity** | | **AMF growth index** | |
| Sobs | 0.83*** | MC | 0.91*** |
| Ace | 0.73*** | HLD | 0.91*** |
| Chao | 0.76*** | Cumulative (%) | 91.11 |
| Shannon | 0.32** | **GRSP** | |
| Cumulative (%) | 66.19 | T-GRSP | 0.94*** |
| **Stability of soil aggregates** | | EE-GRSP | 0.94*** |
| MWD | 1.00*** | Cumulative (%) | 93.90 |
| GMD | 1.00*** |  |  |
| Cumulative (%) | 99.59 |  |  |

**Supplementary Table 4.** The effects of climate change (CC), land-use conversion (LUC) and their interaction (CC × LUC) on AMF diversity, network complexity, AMF growth parameters, GRSP and soil aggregate stability.

| Indices | | CC | | LUC | | CC × LUC | |
| --- | --- | --- | --- | --- | --- | --- | --- |
|  |  | F | p | F | p | F | p |
| AMF diversity | Sobs | 15.06 | **<0.001** | 26.56 | **<0.001** | 0.501 | 0.612 |
|  | Ace | 33.35 | **<0.001** | 40.55 | **<0.001** | 1.18 | 0.325 |
|  | Chao1 | 18.19 | **<0.001** | 20.23 | **<0.001** | 0.07 | 0.933 |
|  | Shannon | 3.85 | **0.036** | 54.71 | **<0.001** | 0.14 | 0.867 |
| AMF network complexity | Number of links | 27.16 | **<0.001** | 4.47 | **0.045** | 0.80 | 0.462 |
|  | Degree | 3.84 | **0.036** | 3.66 | 0.068 | 0.61 | 0.549 |
|  | Clustering coefficient | 55.03 | **<0.001** | 14.12 | **0.001** | 6.99 | **0.004** |
|  | Closeness centrality | 16.04 | **<0.001** | 20.18 | **<0.001** | 3.99 | **0.032** |
|  | Betweenness centrality | 9.37 | **0.001** | 9.68 | **0.005** | 4.61 | **0.020** |
| AMF growth parameters | MC | 77.31 | **<0.001** | 21.09 | **<0.001** | 3.59 | **0.043** |
|  | HLD | 345.7 | **<0.001** | 97.67 | **<0.001** | 20.07 | **<0.001** |
|  | SPD | 0.579 | 0.568 | 0.597 | 0.447 | 3.056 | 0.066 |
| GRSP | T-GRSP | 162.0 | **<0.001** | 219.0 | **<0.001** | 6.47 | **<0.001** |
|  | EE-GRSP | 41.50 | **<0.001** | 135.8 | **<0.001** | 0.803 | 0.460 |
| Soil aggregate stability | MWD | 124.8 | **<0.001** | 232.7 | **<0.001** | 1.33 | 0.284 |
|  | GMD | 150.3 | **<0.001** | 282.9 | **<0.001** | 0.262 | 0.771 |

MC, mycorrhizal colonization; HLD, hyphal length density; SPD, spore density;

**Supplementary Table 5.** Goodness-of-fit statistics (R^2^) for factors fitted to the two dimensional constrained analysis of principal coordinates (2D-CAP) ordination of AMF community structure.

|  | CAP1 | CAP2 | R^2^ | P |
| --- | --- | --- | --- | --- |
| MAT | -0.975 | -0.222 | 0.232 | **0.038** |
| MAP | -0.997 | 0.082 | 0.417 | **0.002** |
| LUC | 0.718 | -0.696 | 0.079 | 0.314 |
| pH | -0.640 | 0.768 | 0.221 | **0.028** |
| TN | 0.998 | 0.064 | 0.367 | **0.004** |
| TP | 0.902 | -0.432 | 0.545 | **0.001** |
| SOC | 0.974 | 0.227 | 0.370 | **0.003** |
| AN | -0.923 | 0.384 | 0.070 | 0.384 |
| AP | -0.901 | -0.434 | 0.236 | **0.030** |
| NO_3_^-^-N | -0.056 | 0.998 | 0.005 | 0.928 |
| NH_4_^+^-N | -0.970 | 0.242 | 0.191 | 0.052 |
| DOC | -0.575 | -0.818 | 0.213 | **0.043** |

MAT = mean annual temperature; MAP = mean annual precipitation; SOC = soil organic carbon; AN = available nitrogen; AP = available phosphorus; TP = total phosphorus; TN = total nitrogen. NO_3_^-^-N = nitrate nitrogen; NH_4_^+^-N = ammonium nitrogen.

**Supplementary Table 6.** Topological features of empirical AMF community networks and comparison with corresponding random networks.

| **Network topological features** | | **Cropland** | | | | **Fallow-land** | | | |
| --- | --- | --- | --- | --- | --- | --- | --- | --- | --- |
|  |  | Cropland | Study sites | | | Fallow-land | Study sites | | |
|  |  |  | HLJ | JL | LN |  | HLJ | JL | LN |
| Empirical  networks | Nodes | 184 | 87 | 111 | 112 | 138 | 120 | 95 | 101 |
|  | Links | 1616 | 260 | 363 | 472 | 713 | 393 | 325 | 536 |
|  | Positive links | 1340 | 213 | 280 | 394 | 531 | 296 | 219 | 397 |
|  | Negative links | 276 | 47 | 83 | 78 | 182 | 97 | 106 | 139 |
|  | % Positive links | 82.92% | 81.92% | 77.13% | 83.47% | 74.47% | 75.32% | 67.38% | 74.07% |
|  | Average degree | 17.565 | 5.971 | 6.541 | 8.429 | 10.333 | 6.550 | 6.842 | 10.641 |
|  | Modularity | 0.718 | 0.964 | 0.950 | 0.714 | 1.017 | 1.095 | 1.304 | 0.814 |
|  | Clustering coefficient | 0.595 | 0.752 | 0.732 | 0.842 | 0.593 | 0.725 | 0.725 | 0.723 |
|  | Network diameter | 6 | 13 | 12 | 10 | 9 | 11 | 12 | 8 |
|  | Graph density | 0.096 | 0.070 | 0.059 | 0.0768 | 0.075 | 0.055 | 0.073 | 0.106 |
|  | Average path length | 2.927 | 4.890 | 4.081 | 3.739 | 3.583 | 4.533 | 3.878 | 2.703 |
| Random  networks | Clustering coefficient | 0.096±  0.003 | 0.069±  0.011 | 0.059±  0.018 | 0.076±  0.008 | 0.075±  0.005 | 0.055±  0.008 | 0.073±  0.009 | 0.106±  0.006 |
|  | Modularity | 0.172±  0.008 | 0.319±  0.021 | 0.310±  0.018 | 0.265±  0.015 | 0.237±  0.012 | 0.311±  0.014 | 0.296±  0.019 | 0.225±  0.014 |
|  | Average path length | 2.071±  0.003 | 2.666±  0.020 | 2.693±  0.015 | 2.432±  0.008 | 2.355±  0.005 | 2.735±  0.015 | 2.557±  0.014 | 2.185±  0.006 |
|  | Network diameter | 3.018±  0.133 | 5.109±  0.381 | 5.103±  0.327 | 4.155±  0.362 | 4.017±  0.130 | 5.106±  0.321 | 4.767±  0.444 | 3.901±  0.287 |
|  | Graph density | 0.096 | 0.070 | 0.060 | 0.076 | 0.075 | 0.055 | 0.073 | 0.103 |
